# Formation of Re-Aggregated Neonatal Porcine Islet Clusters Improves *In Vitro* Function and Transplantation Outcome

**DOI:** 10.3389/ti.2022.10697

**Published:** 2022-12-22

**Authors:** M. Honarpisheh, Y. Lei, Y. Zhang, M. Pehl, E. Kemter, M. Kraetzl, A. Lange, E. Wolf, L. Wolf-van Buerck, J. Seissler, the VANGUARD Consortium, Kevin Bellofatto

**Affiliations:** ^1^ Medizinische Klinik und Poliklinik IV, Diabetes Zentrum - Campus Innenstadt, Klinikum der Ludwig-Maximilians-Universität München, Munich, Germany; ^2^ Chair for Molecular Animal Breeding and Biotechnology, Gene Centre and Department of Veterinary Sciences, Ludwig-Maximilians-Universität München, Munich, Germany; ^3^ German Center for Diabetes Research (DZD), Neuherberg, Germany

**Keywords:** islet transplantation, xenotransplantation, neonatal islet-like cell clusters, re-aggregated cell clusters, porcine islets, pseudo-islets

## Abstract

Neonatal porcine islet-like cell clusters (NPICCs) are a promising source for islet cell transplantation. Excellent islet quality is important to achieve a cure for type 1 diabetes. We investigated formation of cell clusters from dispersed NPICCs on microwell cell culture plates, evaluated the composition of re-aggregated porcine islets (REPIs) and compared *in vivo* function by transplantation into diabetic NOD‐SCID IL2rγ^−/−^ (NSG) mice with native NPICCs. Dissociation of NPICCs into single cells and re-aggregation resulted in the formation of uniform REPI clusters. A higher prevalence of normoglycemia was observed in diabetic NSG mice after transplantation with a limited number (*n* = 1500) of REPIs (85.7%) versus NPICCs (*n* = 1500) (33.3%) (*p* < 0.05). Transplanted REPIs and NPICCs displayed a similar architecture of endocrine and endothelial cells. Intraperitoneal glucose tolerance tests revealed an improved beta cell function after transplantation of 1500 REPIs (AUC glucose 0–120 min 6260 ± 305.3) as compared to transplantation of 3000 native NPICCs (AUC glucose 0–120 min 8073 ± 536.2) (*p* < 0.01). Re-aggregation of single cells from dissociated NPICCs generates cell clusters with excellent functionality and improved *in vivo* function as compared to native NPICCs.

## Graphical Abstract

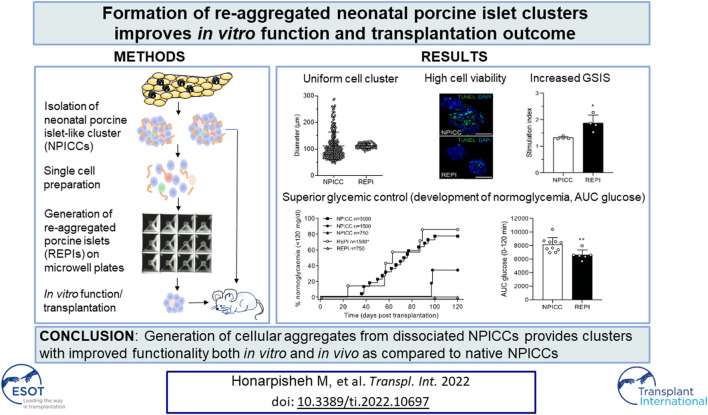

## Introduction

Islet cell transplantation represents a promising therapy to achieve normoglycemia in patients with type 1 diabetes (1). Loss of islet mass during the isolation process and in the first days after transplantation is a major challenge for islet transplantation. This is mainly mediated by hypoxia due to the limitation of oxygen diffusion until sufficient revascularization has developed (2, 3). It has been shown that smaller islets display a better function *in vitro* and *in vivo* (4-6). Native mouse and human islets have been reported to spontaneously re-aggregate into cell clumps after being dissociated into single cells (7). Pre-defined uniform cluster size was achieved using the hanging drop culture or customized microwell devices (8-12).

Since the supply of high-quality human islets is limited by the paucity of organ donors, alternative cell sources such as porcine islet cells are demanded. Xenotransplantation of pig islets is very promising because supply of porcine islets is unlimited, techniques to isolate islets on a large scale are established (13), and recent progress on genetic modification of pigs has generated donor animals which provide islets which significantly decrease the severity of humoral and cellular immune responses (14). Successful long-term transplantation of neonatal and adult porcine islets into diabetic non-human primates (NHP) with insulin independence for a maximum of 965 days were described in several studies under systemic immunosuppression using potent co-stimulation inhibitors (15-17). However, translation of these studies to clinical trials is still limited because antibodies to block the CD40/CD154 costimulation pathway are not approved for application in human beings thus far (18).

Neonatal porcine islet like cell clusters (NPICCs) represent useful candidate cells for xenotransplantation (19) because they are easier to isolate as compared to adult islets and more robust against hypoxia and inflammation (20). The disadvantage is that NPICCs consist of immature cells and precursor cells which need several weeks for maturation after transplantation until glucose-dependent insulin secretion and normoglycemia have developed (13, 21). The requirement of a high number of islets to correct hyperglycemia after transplantation together with the lower yield from neonatal as compared to adult pig pancreas demands to increase either the isolation or the transplantation efficacy.

In the present study we investigated whether islet cell function can be improved by dissociation of NPICCs and re-aggregation into uniform cell clusters.

## Materials and Methods

### Animals

German landrace hybrid piglets served as pancreas donors. NOD-SCID IL2rγ^−/−^ (NSG) mice, which lack mature T cells, B cells and NK cells, were obtained from The Jackson Laboratory (strain 005557) and housed under standard SPF conditions. All animal experiments were approved by the responsible authority and performed in agreement with the German Animal Welfare Act and Directive 2010/63/EU.

### Islet Isolation and Generation of Dissociated Single Cells

NPICCs were isolated from pancreata (*n* = 12) of 2–5 days-old piglets by collagenase digestion as described previously (22, 23). Cell clusters were cultured in RPMI 1640 (PAN-Biotech, Aidenbach, Germany), 2% human serum albumin (Takeda, Konstanz, Germany), 10 mM nicotinamide, 20 ng/ml exendin-4 (Merck, Darmstadt, Germany) and 1% antibiotic-antimycotic (Thermo Fisher Scientific, Germering, Germany) (basal islet culture [B-IC] medium). On day 4, NPICCs were harvested and islet equivalents (IEQ) were determined under a stereomicroscope. NPICCs were either re-cultivated in B-IC medium (control group) or washed with PBS and incubated in TrypLE Express solution (Thermo Fisher Scientific) for 8–12 min at 37°C with gentle mixing every 60 s until dispersion into single cells was observed under a stereomicroscope. Then, the cells were filtered through a 40-µm filter (Corning, Wiesbaden, Germany) to remove debris.

### Formation of Re-aggregated Islet Cells

Dispersed islet cells were selected at random and seeded on Sphericalplate 5D (Kugelmeiers, Erlenbach, Switzerland), which contain 750 microcavities per well, in 2 ml B-IC medium to yield re-aggregated clusters composed of 750 cells. This cell number was chosen from previous studies reporting on an optimal islet aggregate size of about 100 µm (6, 10, 24, 25). Plates were centrifuged at 250xg for 3 min and incubated in B-IC medium for 3 days. Then, clusters were gently flushed out from the wells, washed with medium or buffer, and used for measurement of cell viability, glucose stimulated insulin secretion (GSIS), and transplantation.

### Islet Cell Viability, Composition, and Recovery

Cell viability was detected by calcein AM (live cells) and propidium iodide (dead cells) dye staining according to the manufacturer´s instruction (Thermo Fisher Scientific). Samples were analyzed under a fluorescent microscope (n > 50 cluster) and by flow cytometry at day 7 after isolation. To determine the cluster architecture, NPICCs and REPIs were embedded in Epredia™ HistoGel™ Specimen Processing Gel (Thermo Fisher Scientific) and stained for insulin (guinea pig anti-insulin, 1:400, Agilent-Dako, Frankfurt, Germany) and glucagon (rabbit anti-glucagon, 1:100, Cell Signaling, Frankfurt, Germany) followed by incubation with FITC-labelled anti-rabbit IgG and Cy3-labeled anti-guinea pig IgG (Thermo Fisher Scientific). DAPI was used to counterstain cell nuclei. Recovery rate was determined by calculation of the ratio IEQ of REPIs to IEQ of native NPICCs. Apoptotic cells were analyzed on day 7 by TUNEL staining of NPICCs and REPIs using the DeadEnd Fluorometric TUNEL System assay according to the manufacturer´s instructions (Promega, Madison, WI, United States).

### Static Glucose-Stimulated Insulin Secretion

On day 7 after isolation, 100–150 islet equivalent (IEQ) of NPICCs and REPIs were washed in glucose-free RPMI medium and Krebs-Ringer buffer (KRB) solution and pre-incubated in KRB containing 2.8 mmol/L glucose (low glucose) for 1 h at 37°C and 5% CO_2_ followed by incubation in duplicates in KRB with low glucose or high glucose (20.0 mmol/L) for 1 h. Then, supernatants were collected and porcine insulin concentration was measured in duplicates by ELISA (Mercodia, Uppsala, Sweden). The stimulation index (SI) was calculated by dividing the insulin concentration in high glucose by insulin concentration in low glucose (23).

### Transplantation

After induction of diabetes by intraperitoneal injection of 180 mg/kg body weight streptozotocin, diabetic NSG mice (blood glucose levels >350 mg/dl) received native NPICCs (3000, 1500, or 750 IEQs/mouse) or REPIs (750 or 1500 IEQs/mouse) under the left kidney capsule as described recently (22, 26). 3000 IEQs represent the standard dose of NPICCs for transplantation to achieve normoglycemia in about 80% of diabetic NSG mice. Blood glucose levels were monitored by FreeStyle Lite blood glucose test strips (Abbott, Wiesbaden, Germany). Diabetic mice with blood glucose levels >300 mg/dl were treated with insulin glargine (0.25–1 IE s.c. daily). Our primary endpoint was normoglycemia. The observation period was set to a maximum of 16 weeks. We used a stringent definition for normoglycemia which was specified as achievement of pretransplant glycemia (persistent random non-fasting blood glucose levels <120 mg/dl). This cut-off is based on the measurement of non-fasting blood glucose levels of untreated NSG mice (*n* = 107; 94.6 ± 15.2 mg/dl, range 62–128 mg/dl). In this control cohort there was no animal with non-fasting blood glucose levels above 120 mg/dl on two consecutive days. Because other studies often used non-fasting blood glucose levels <180 mg/dl to define normoglycemia after islet transplantation this threshold was also analyzed.

### Characterization of Graft Function

Glucose tolerance in transplanted mice was determined by intraperitoneal glucose tolerance test (IPGTT) 10–14 days after development of normoglycemia using 2 g glucose/kg body weight. Blood samples were obtained from the tail vein at 0 and 10 min to measure porcine insulin in duplicates by ELISA (Mercodia) that had no cross-reactivity with mouse insulin. To provide evidence that normoglycemia was mediated by the grafted tissue and not by pancreatic islet regeneration, graft bearing kidneys were removed in three transplanted animals followed by daily blood glucose measurements for 3 days. When the post uninephrectomy blood glucose level was >400 mg/dl, the achieved normoglycemia was considered as graft-dependent.

### Cellular and Morphological Characteristics Analyzed by Immunohistochemistry

Paraffin sections of the graft bearing kidney were stained with the following antibodies (Ab): guinea pig anti-insulin (1:400, Agilent-Dako), rabbit anti-glucagon (1:100, Cell Signaling), rabbit anti-somatostatin (1:50, Agilent-Dako), rabbit anti-pancreatic polypeptide (PP) (1:5000, Proteintech) and rabbit anti-CD31 (1:100, Cell Signaling). Secondary antibodies used were HRP- or alkaline phosphatase-conjugated anti-guinea pig IgG (Agilent-Dako) and anti-rabbit IgG (Vector Laboratories, California, United States). Fuchsin + substrate chromogen (Agilent-Dako) or 3,39-diaminobenzidine (Kem-En-Tec Nordic A/S, Uppsala, Sweden) were used as chromogen. To visualize glucagon and PP, the ImmPRESS® HRP horse anti-rabbit IgG polymer detection kit (Vector Laboratories) was used.

Cellular composition of NPICCs and REPIs were quantified by QuPath software (version 0.3.2) using pictures scanned by uScope MXII slide scanner (Microscope International, Dallas, United States). The numbers of cells that stained positive for glucagon, somatostatin or PP were expressed as number of positive cells per 100 insulin positive beta cells. To assess vascularization, the area of CD31 positive cells (endothelial cell marker) were detected and normalized to the islet area.

### Flow Cytometry

Accuri C6 flow cytometer (BD Biosciences, Heidelberg, Germany) was used to determine the number of insulin-, glucagon-, and somatostatin-positive cells in NPICCs and REPIs. Single cells were prepared by digesting cell clusters with TrypLE solution (Thermo Fisher Scientific), washed with PBS +10% fetal calf serum (FCS), and filtered through a 30 µm pre-separation filter (Miltenyi, Bergisch-Gladbach, Germany). Then, cells were fixed/permeabilized with an intracellular staining buffer set (Thermo Fisher Scientific) and incubated with Fc-Block (anti-mouse CD16/CD32) for 10 min at room temperature. Thereafter, cells were stained with fluorochrome-labeled antibodies against insulin (anti-insulin-AF647, clone T56-706), glucagon (anti-glucagon-PE, clone U16-850), and somatostatin (anti-somatostatin-AF488, clone U24-354) (BD Biosciences). All antibodies were pretested for appropriate dilution and specificity using isotype control antibodies. Antibodies were incubated at 4°C for 30 min, washed two times with permeabilization buffer and analyzed on a flow cytometer with FlowJo software version 10.4 (TreeStar, Ashland, United States).

### Statistical Analysis

Data are presented as means and standard deviations (SD). Statistical differences between two groups were analyzed with Shapiro-Wilk test for normality and F test for homogeneity of variance and examined with the Mann-Whitney *U* test. Time to normoglycemia was compared with log-rank test. The area under the curve (AUC) was calculated using trapezoidal rules. *p* values <0.05 were considered statistically significant. Statistical analyses were performed using GraphPad Prism (version 9.2, GraphPad, San Diego, United States).

## Results

### Re-Aggregated Cells Show Uniform Size and Improved *In Vitro* Function

**FIGURE 1:**
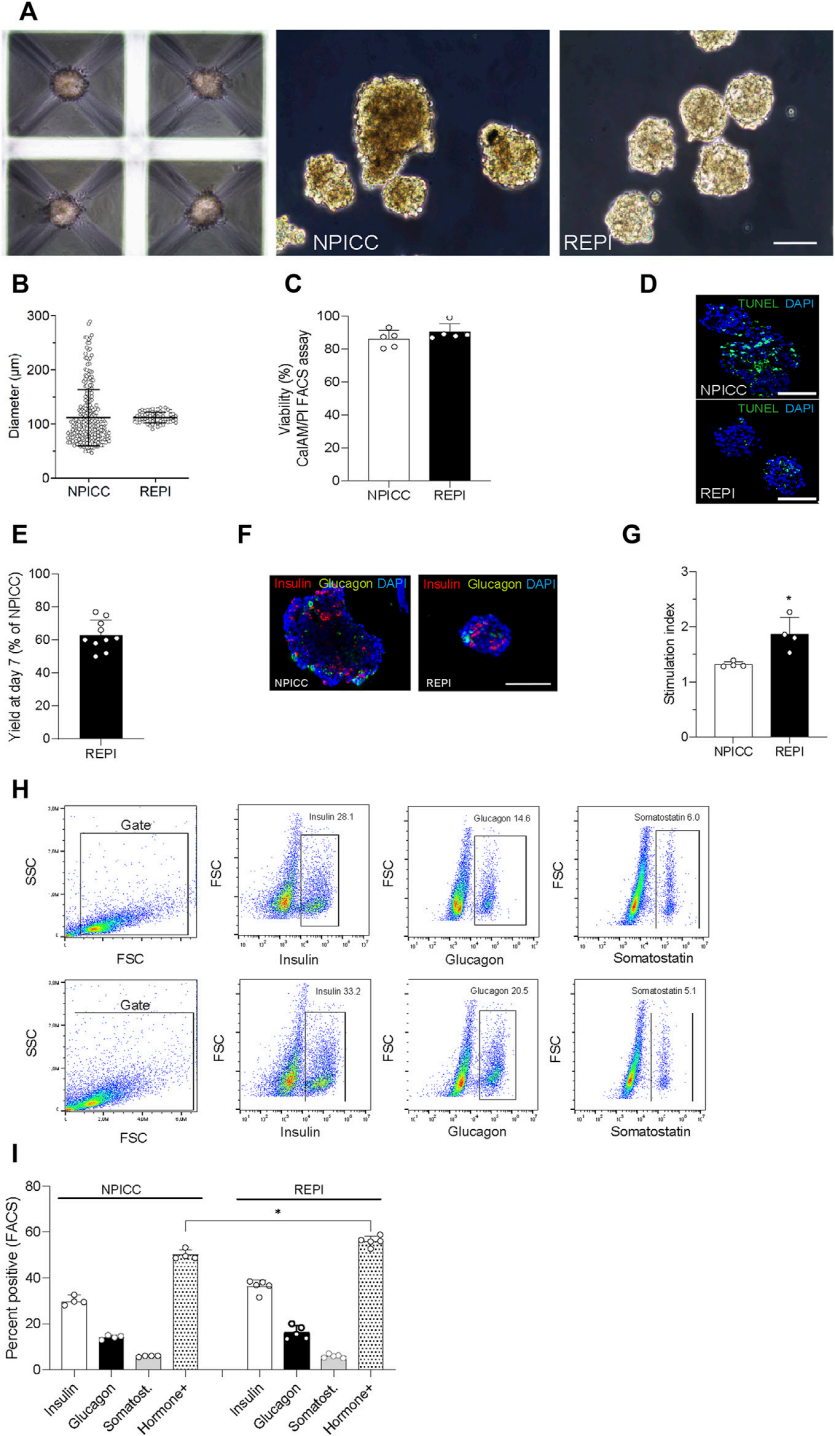
Phenotype and architecture of native neonatal porcine islet-like clusters (NPICCs) and re-aggregated porcine islets (REPIs) on day 7 after isolation. **(A)** Phase contrast pictures of cells cultured in Sphericalplate 5D with 750 cells per microwell (left) and morphology of NPICCs (middle) and REPIs (right) after harvesting. Scale bars, 100 µm. **(B)** Size distribution of REPIs and NPICCs from *n* = 5 pancreata. Box plots with median diameter, 25th-75th percentile and minimum and maximum (whiskers). **(C)** Viability of cells analyzed by FACS (right) and fluorescence microscopy (left) using calcein AM (green, live cells) and propidium iodide (PI) (red, dead cells) dye staining (*n* = 5). **(D)** TUNEL assay revealed many TUNEL positive cells (green) in the inner core of large NPICCs. Scale bars, 100 µm **(E)** Recovery rate of REPIs as percentage of NPICCs (n = 10). **(F)** Immunofluorescence staining of insulin (red) and glucagon (green) in NPICCs and REPIs (*n* = 4). Scale bars, 100 µm. **(G)** Measurement of *in vitro* beta cell function assessed by glucose stimulated insulin secretion (*n* = 4). **(H)** Representative flow cytometric characterization of insulin, glucagon and somatostatin positive cells. **(I)** Analysis of the percentage of insulin (white bars), glucagon (black bars) and somatostatin (grey bars) positive cells and sum of hormone positive cells (dotted bars) (*n* = 4). Data are presented as mean ± SD. *p < 0.05, **p < 0.01 vs. NPICCs.

On day 3 after isolation, NIPPCs were dissociated by mild TypLE treatment resulting in single cells with a viability of 88.2 ± 5.6%. After 3–4 days of seeding in 3D-cluture plates, clusters composed of 750 re-aggregated cells showed a uniform size with a mean diameter of 113.3 ± 10.5 µm (range 94.8–130.4, median 113.4 µm) whereas the diameter of native NPICCs was much more heterogeneous (range 50.1–289.6 µm, median 95.2 µm) (Figures 1A–C). The size variation of native NPICCs was significantly higher as compared to REPIs (*p* < 0.01). As expected, there was cell loss during the cell dissociation and re-aggregation process. The recovery rate, defined as IEQ of REPIs compared to control NPICCs on day 7 after isolation was 63.5 ± 18.9% (Figure 1D).

Fluorescence microscopy revealed that insulin-positive cells were more uniformly dispersed and their relative abundance was increased in REPIs as compared to NPICCs (Figure 1E). This was confirmed by flow cytometry where slightly higher percentage of insulin- and glucagon-positive cells were detected. The proportion of somatostatin-positive cells was not altered. The percentage of endocrine cells (sum of alpha, beta and delta cells) was significantly increased in re-aggregated clusters (55.9 ± 2.2% vs. 50.0 ± 2.2% in NPICCs) (*p* < 0.05) (Figure 1F).

Glucose-dependent insulin secretion was assessed in four independent experiments with handpicked NPICCs and REPIs. REPIs exhibited a significantly higher GSIS (1.9-fold) as compared to native NPICCs (1.4-fold) (*p* < 0.05) (Figure 1G). Taken together, compared to conventional NPICCs, REPIs had a higher homogeneity in size, contained higher proportions of endocrine cells, and could serve as a suitable source for *in vivo* transplantation.

### REPIs Display Improved Function After Transplantation

**FIGURE 2:**
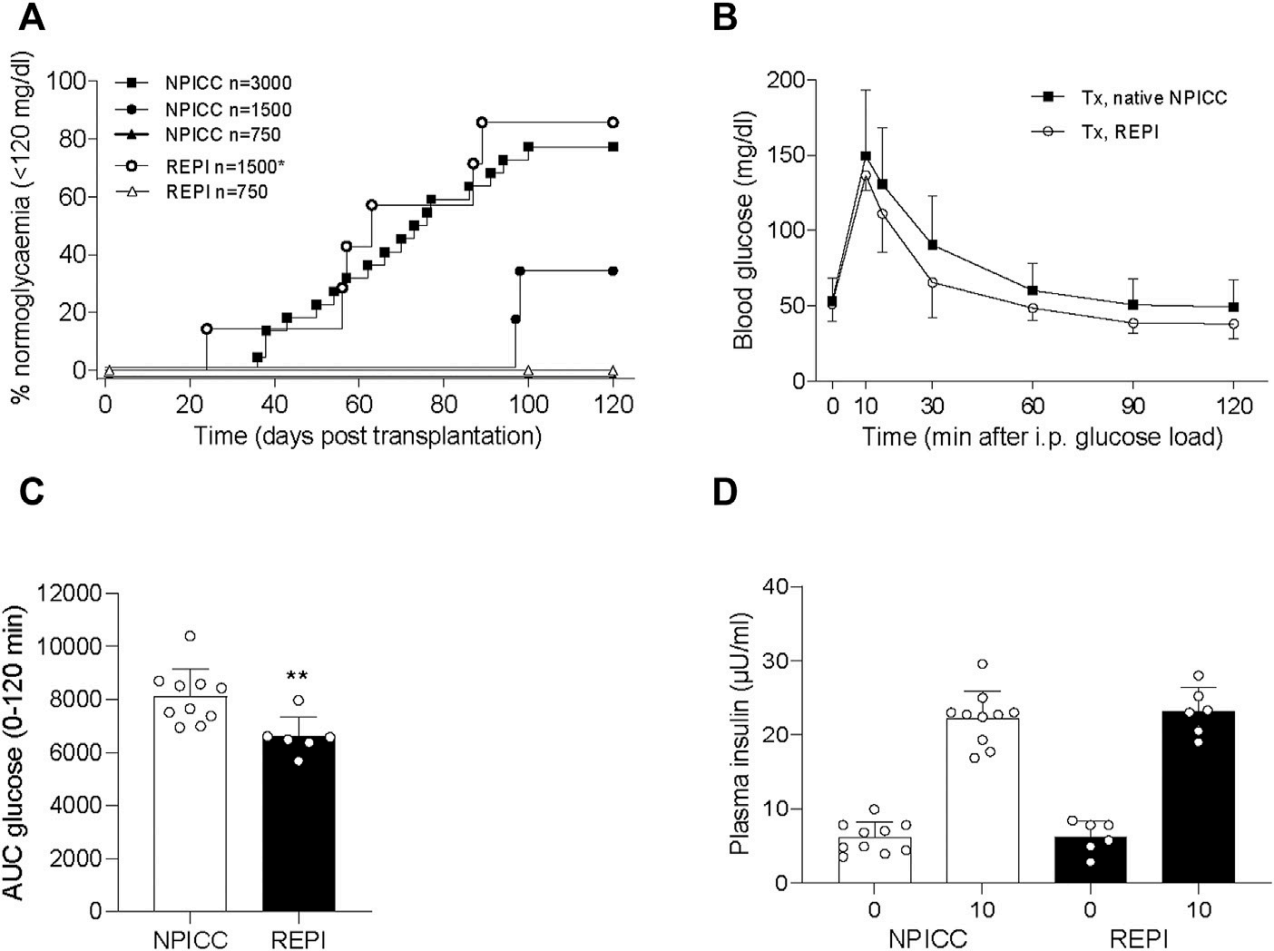
Transplantation with REPIs improved reversal of diabetes. **(A)** Kaplan-Meier analysis of time to develop normoglycemia in diabetic NSG mice transplanted with 750 (*n* = 4) and 1500 REPIs (*n* = 7) or 750 (*n* = 4), 1500 (*n* = 6) and 3000 NPICCs (*n* = 22) on day 7 after isolation. After transplantation with 1500 REPIs significantly more animals developed normoglycemia defined by non-fasting blood glucose <120 mg/dl compared to the group transplanted with 1500 NPICCs (*p < 0.05). **(B–D)** Intraperitoneal glucose tolerance test (IPGTT) in diabetic NSG mice transplanted with 1500 REPIs (*n* = 6) or 3000 NPICCs (*n* = 10). **(B)** Glucose response curve during the IPGTT. **(C)** Glucose clearance during IPGTT assessed by calculating area under the curve (AUC) for glucose 0–120 min was improved in animals transplanted with REPIs (*n* = 6) **p < 0.01 vs. NPICC group. **(D)** Insulin secretion at 0 and 10 min after glucose challenge was similar in both transplantation groups. Data are presented as the mean ± SD.

We next validated the *in vivo* function of REPIs in diabetic NSG mice by transplanting 750 and 1500 IEQ REPIs. We included groups transplanted with 750, 1500, and 3000 IEQ native NPICCs as controls. All animals remained diabetic after transplantation of 750 native NPICCs or 750 REPIs (Figure 2A). Prevalence of non-fasting blood glucose levels <120 mg/dl was 85.7% in mice transplanted with 1500 IEQ REPIs (*n* = 6 of 7) (median diabetes reversal time 63 days) as compared to around 33% (*n* = 2 of 6) in those transplanted with 1500 native NPICCs (median diabetes reversal time 98 days) (*p* < 0.05) (Figure 2A). Notably, the reversal rate and median diabetes reversal time of mice transplanted with 1500 REPIs (85.7% 63 days) were comparable to those of mice with 3000 native NPICCs (77.2%; 66 days), indicating a higher efficacy per IEQ unit for REPIs.

Similar results were obtained by using blood glucose levels <180 mg/dl as the threshold for normoglycemia. Percentage of mice below this cut-off was significantly higher in animals transplanted with 1500 IEQ REPIs (85.7%, median time 59 days) versus 1500 native NPICC (50%, median time 90 days) (*p* < 0.05) (Supplementary Figure S1).

We further performed IPGTT in mice developing normoglycemia. As shown in Figure 2C, the glucose clearance in the group that received 1500 REPIs (AUC 6260 ± 305.3) was significantly better than that in the group with 3000 NPICCs (AUC 8073 ± 536.2) (*p* < 0.01). Plasma insulin levels at 0 and 10 min during IPGTT were similar in all groups (Figure 2D). These data suggested that the re-aggregation of clusters from dispersed NPICCs resulted in a significant improvement of *in vivo* function.

### Architecture of Re-Aggregated Clusters

**FIGURE 3:**
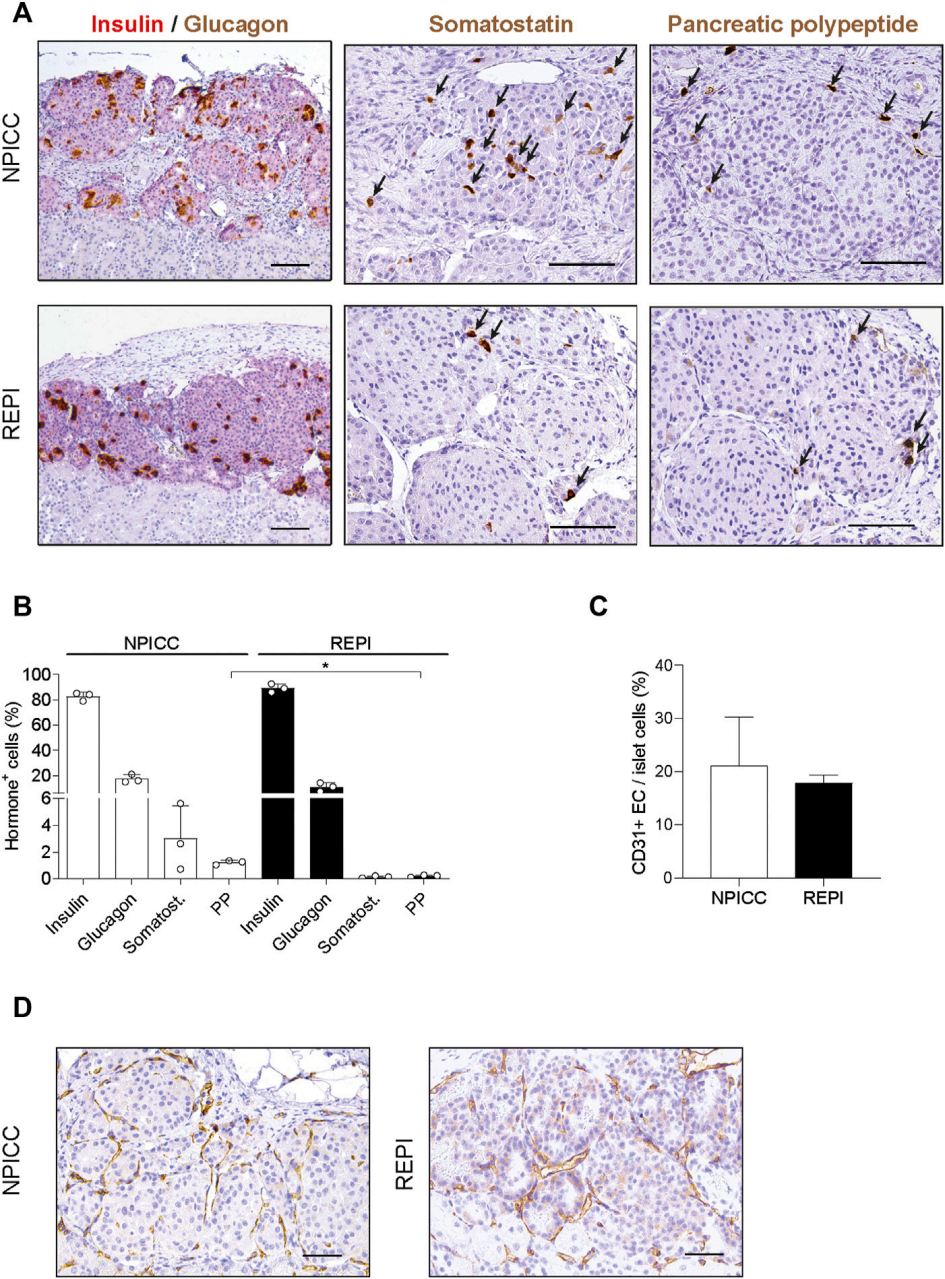
Architecture and revascularization of transplanted grafts. **(A)** Representative immunohistochemical staining for insulin (red)/glucagon (brown), somatostatin (brown, arrows) and pancreatic polypeptide (PP) (brown, arrrows) in sections of grafted NPICCs and REPIs (1500 IEQ). **(B)** Quantification of endocrine cells within the grafts revealed a similar proportion of insulin, glucagon and somatostatin cells and a significant lower frequency of PP cells in grafted REPIs (*n* = 3 per group). **p < 0.01. **(C)** Quantification of CD31 positive endothelial cells (EC) by immunohistochemistry (*n* = 3 per group). The results are expressed as CD31 positive area normalized to islet area. **(D)** Representative images of CD31 staining in grafts. Characteristic CD31 staining (brown) of blood vessels in the grafts. Data are represented as the mean ± SD. Scale bars, 100 µm.

To characterize the composition of endocrine cells and the density of vascularization following transplantation with NPICCs and REPIs, immunohistochemistry was performed at the end of the post-transplantation period. Three grafts per group were stained to detect endocrine cell components including insulin, glucagon, somatostatin and pancreatic polypeptide (PP) positive cells. Although there was a random rearrangement of endocrine cells in the first days after re-aggregation, this picture changed during *in vivo* maturation. As shown in Figure 3, the grafts derived from REPIs and from NPICCs consisted of a core of insulin-positive cells, surrounded by glucagon-, somatostatin- and PP-positive cells, which mainly located in the islet mantle (Figures 3A,B), suggesting the spatial reorganisation of endocrine cells *in vivo* during the post-transplant period.

There were small differences in the morphology between REPI and NPICC grafts. The relative proportion of insulin-positive cells in REPIs was slightly higher than in NPICC grafts (89.1 ± 3.4% vs. 82.6 ± 3.2%) . Conversely, glucagon- (10.9 ± 3.3% vs. 17.3 ± 3.2%) and somatostatin- (0.2 ± 0.1% vs. 2.3 ± 0.1%) positive cells were slightly decreased while the relative proportion of PP-positive cells was significantly reduced in REPI grafts (0.2 ± 0.1% vs. 1.2 ± 0.2%) as compared to NPICC grafts from the same pig donors (*p* < 0.01). (Figure 3B). Quantification of CD31 staining within the grafts revealed no difference in endothelial cell (EC) area in both groups (Figures 3C,D).

## Discussion

This study provides evidence that the *de novo* formation of uniform cell clusters from neonatal porcine islets in 3D-culture plates has the potential to generate pseudo-islets with a higher proportion of endocrine cells, an enhanced *in vitro* function and an improved transplantation outcome than native NPICCs.

The study was initiated to evaluate the performance of REPIs in comparison to NPICCs originated from the same donor animals. For the first time we were able to show that neonatal porcine islets, which are mainly composed of immature endocrine cells and progenitor cells, benefit from single-cell preparation and re-aggregation in a similar way as reported from studies using pseudo-islets derived from dissociated adult human and rat islets. This includes an improved *in vitro* glucose responsiveness and a changed pattern of endocrine cell distribution of the gravity-mediated, newly formed cell clusters as compared to native islets (6, 11, 25, 27). In large NPICCs, we frequently observed a dark core with absent DAPI nuclear stain suggesting dead cells or components of extracellular matrix which increased by time of culture. By single cell preparation these dead cells can be eliminated. After formation of weakly aggregated size-controlled clusters the diffusion of oxygen and essential nutrients may be accelerated (7, 25, 28). This improved supply may be one explanation why REPIs had a significantly higher percentage of endocrine cells and an increased *in vitro* insulin secretion capacity in response to high glucose.

The number of high-quality islets and the lag time to reach a full functioning of beta cells due to hormonal immaturity remains a major problem in NPICC transplantation. The most important question of the present study was whether the preparation of REPIs enables to improve development of normoglycemia after transplantation into diabetic mice. We used the marginal islet mass transplant model to test the ability of REPIs to reverse hyperglycemia. 1500 REPIs performed significantly better than 1500 native NPICCs and achieved diabetes reversal in a similar time frame as after transplantation of 3000 NPICCs. Improved outcome after transplantation of a limited number of re-aggregated rat and human islets as compared to control islets was described in several studies (6, 7, 11, 12). Thus, REPIs clearly provide an advantage over native NPICCs. In part, this may be due to a better hypoxia tolerance in the first hours after transplantation mediated by the increased oxygen tension due to a lower diffusion distance in comparison to the large NPICCs. Small native and re-aggregated human and rat islets have been shown to undergo a less sustained period of hypoxia and to develop a reduced necrotic core after transplantation underneath the kidney capsule or into subcutaneous tissue (4, 5, 7, 11). By immunohistochemistry a decreased number of PP cells as well as a trend towards a lower alpha and delta cell frequency was detected at the end of the observation period. Lower intraislet somatostatin and glucagon secretion may be another explanation for the significantly increased glucose clearance during IPGTT in mice transplanted with REPIs. Previous studies have shown that re-aggregated pseudo-islets develop a higher vessel density because they are more easily penetrated by newly forming capillaries (12). We detected similar volumes of CD31-positive endothelial cells in the grafts suggesting no significant differences in vascularization between both transplantation groups.

One important problem of the preparation of REPIs is the cell loss observed during single cell production and re-aggregation culture. To improve cell recovery from isolated NPICCs we performed gentle cell dissociation under visual controls to avoid over-digestion, rapid cell washing steps and centrifugation-forced cluster formation on 3D-culture plates. Nevertheless, the calculated recovery rate of REPIs in relation to NPICCs was only 63.5%. This cell yield seems to be low, but is in the range of the 50%–90% cell loss described in most studies using dissociated adult human and rat islets (4, 6, 7). The low cell yield may only partly explained by the cell dissociation process itself, because the cell viability was similar in REPIs and NPICCs. The elimination of damaged and irrelevant non-endocrine or inflammatory cells and the removal of extracellular matrix from the inner core of the islets may be one important factor for the observed reduction of REPI volume resulting in a decreased IEQ ratio. Considering not only the recovery ratio but also the equal transplantation outcome of 1500 REPIs and 3000 NPICCs, the use of REPIs results in an enhanced efficiency (1.2-fold per organ donor pancreas) for transplantation of diabetic mice with a curative islet dose. Thus, the better performance of REPIs fully compensates for the tissue loss during the dissociation and re-aggregation procedure.

In conclusion, we here demonstrate a simple and efficient method to easily produce re-aggregated clusters composed of neonatal porcine islet cells in a microwell format which has the potential for large scale generation of REPIs. The formation of REPIs has a significant impact on *in vitro* and *in vivo* beta cell function and the outcome of porcine islet transplantation. REPIs may facilitate future studies on NPICC cell manipulation to induce progenitor and beta cell proliferation and differentiation in order to increase pre- and post-transplant beta cell mass. The availability of size-defined clusters represent a substantial improvement for studies aiming to use encapsulated porcine islets. Moreover, it will become possible to fabricate clusters composed of different cell types including endothelial cells and immunomodulatory cells to further improve vascularization and to modulate graft rejection, thereby generating an improved self-protective islet graft. This will be the focus of our studies in the near future.

## VANGUARD Consortium

Kevin Bellofatto, Ekaterine Berishvili, Juliette Bignard, Laura Mar Fonseca, Fanny Lebreton (Department of Surgery, University of Geneva, Geneva, Switzerland), Simone Assanelli, Chiara Borsotti, Alessia Cucci, Antonia Follenzi, Cristina Olgasi (Department of Health Sciences, University of Piemonte Orientale, Novara, Italy), Antonio Citro, Lorenzo Piemonti, Silvia Pellegrini (IRCCS Ospedale San Raffaele, Diabetes Research Institute, Milano, Italy), Olivier Thaunat (Dept. Transplantation, Nephrology and Clinical Immunology, Lyon Claude Bernard University, Lyon, France), Dide de Jongh, Emma Massey (Erasmus MC, University Medical Centre, Rotterdam, Netherlands), Eline Bunnik (Dept. of Medical Ethics, Philosophy and History of Medicine, University Medical Centre, Rotterdam, Netherlands), Antonia J. Cronin (King’s College, London, United Kingdom), Devi Mey, Chiara Parisotto, Giovanna Rossi (European Society for Organ Transplantation, Padova, Italy), Patrick Kugelmeier, Markus Mühlemann, Karolina Pal-Kutas, Petra Wolint (Kugelmeiers AG, Erlenbach, Switzerland), Marco Cavallaro, Julia Götz, Jeanette Müller (Accelopment Switzerland Ltd.).

## Data Availability

The original contributions presented in the study are included in the article/Supplementary Material, further inquiries can be directed to the corresponding author.

## Supplementary Material

Supplementary Figure S1 Analysis of time to develop non-fasting blood glucose levels <180 mg/dl. After transplantation with 1500 REPIs (*n* = 7) significantly more animals developed glycemia below this cut off as compared to animals transplanted with 1500 NPICCs (*n* = 6) (**p* < 0.05).

## Abbreviations

AUC, area under the curve; IPGTT, intraperitoneal glucose tolerance test; NSG, NOD-SCID IL2rγ^−/−^; NPICC, neonatal porcine islet-like cell cluster; REPI, re-aggregated neonatal porcine islet-like cluster.
