# Supplementary figures and images for: Formation of Re-Aggregated Neonatal Porcine Islet Clusters Improves In Vitro Function and Transplantation Outcome
Source: Transpl Int. 2022 Dec 22;35:10697. doi: 10.3389/ti.2022.10697 (PMC9846776; doi:10.3389/ti.2022.10697)

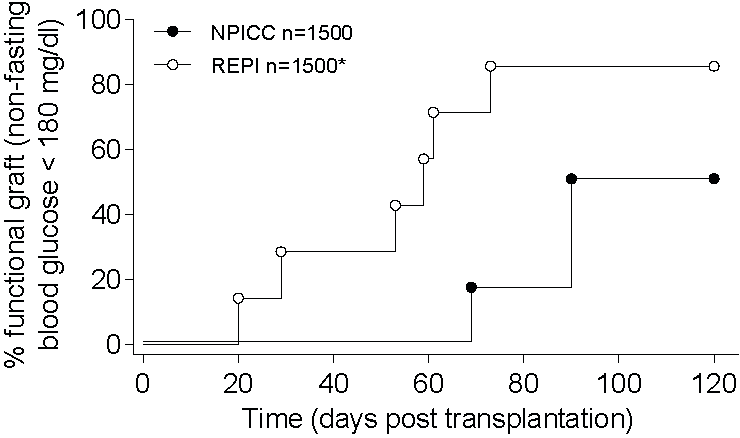

Supplement: Supplementary file 1 [file Image1.tif]
